# The intersections of industry with the health research enterprise

**DOI:** 10.1186/s12961-019-0457-7

**Published:** 2019-05-29

**Authors:** Elie A. Akl, Assem M. Khamis

**Affiliations:** 1 Department of Internal Medicine, American University of Beirut Medical Center, P.O. Box: 11-0236, Riad-El-Solh Beirut, Beirut, 1107 2020 Lebanon; 2 Clinical Research Institute, American University of Beirut Medical Center, Beirut, Lebanon; 3 Department of Health Research Methods, Evidence, and Impact (HEI), McMaster University, Hamilton, Ontario Canada

**Keywords:** Industry, health research, funding

## Abstract

There is increased awareness of the negative impact of large multinational corporations – the ‘industry’ – on public health. These corporations have established different types of relationships with a number of actors in the field of health research. This Commentary explores the different types of relationships between the industry and the actors of health research, how they intersect with the different research steps, and how these relationships allow the industry to exert influence. The types of relationships discussed consist of funding of research, direct relationships with the actors of research (namely advocacy groups, funding agencies, experts, professional organisations, regulatory agencies and health practitioners), and the influencing research standards. The potentially influenced research steps either precede the research (i.e. the prioritisation of research question), relate to it directly (i.e. its planning, conduct, reporting, dissemination and evaluation), or build on it (i.e. regulatory approval, integration into guidelines and adoption into practice). In conclusion, the industry has successfully fostered relationships with almost every actor of the health research enterprise and is using these relationships to influence the different steps of health research. The degree of influence the industry is having on health research calls for more work on managing the relationships discussed herein.

## Introduction

There is increased awareness of the negative impact of large multinational corporations – the ‘industry’ – on public health. Some of these companies prioritise profit, even when that means the marketing of unhealthy products. The tobacco industry, for example, worked on its expansion and marketing in low- and middle-income countries (e.g. in sub-Saharan Africa) to increase tobacco use, especially among women and children [1], as well as on subverting public health policies for tobacco control [2]. In relation to the food sector, there is evidence that sugar consumption increases the risk of stroke [3], rheumatoid arthritis [4], hypertension and obesity [5, 6].

The growing impact of industry on public health has led to the concept of corporate determinants of health [7], sometimes referred to as commercial determinants of health [8]. Kickbusch et al. [9] described four channels through which corporations exert their influence, namely marketing, lobbying, corporate social responsibility strategies and extensive supply chains. Corporations use those channels to impact lifestyle choices and drive consumers’ options in ways that favour their profits. Within that broad picture, the industry may use the output of health research in their marketing and lobbying efforts as well as the cover of corporate social responsibility to fund research. One could also argue that the intersection of the industry with the health research enterprise represents a fifth channel. One has to acknowledge that the intersection between industry and health research could be beneficial, as it can support medical innovations and various research types [10]. In addition, there are instances where the access to industry resources and technology helped health scientists to advance medicine and public health [10].

The objective of this paper is to review the intersections between industry and the health research enterprise that may negatively impact the latter. The paper specifically explores (1) the different types of relationships between the industry and the actors of health research; (2) the different steps of potential influence on health research; and (3) how these relationships allow the industry to exert influence.

We used the term ‘industry’ to refer to the large multinational corporations whose products might have a negative impact on health, such as the tobacco, pharmaceuticals, alcohol, food and beverage industries. We do not address practices that are considered illegal such as monitoring and threatening scientists [11, 12], or practices that are considered unethical, e.g. falsification, gift authorship or ghost authorship.

Box 1 shows the eight types of relationship between the industry and the actors of health research. These types consist of either funding of research, direct relationships with the actors of research (namely advocacy groups, funding agencies, experts, professional organisations, regulatory agencies and health practitioners), and influencing of research standards. This classification is based on our review of the literature, particularly the published frameworks of the types of stakeholders in health research (see Appendix C in Concannon et al.) [13].

Box 2 shows the nine steps of potential influence that the industry exerts on health research. These steps are related to either preparing for the research (i.e. prioritisation of the research question), conducting the research (i.e. its planning, conduct, reporting and dissemination), or its use (i.e. evaluation, regulatory approval, integration into guidelines and adoption into practice). This classification builds on a review of the literature, including a matrix summarising approaches to stakeholder engagement in research [13].

Table 1 consists of a matrix showing the intersection between the types of relationships and the different research steps. Below, we discuss these intersections according to the types of relationships.

**Table 1:** Matrix showing the intersection between the types of relationships and the research steps of potential influence

	Prioritisation of research question	Planning of the study	Conduct of the study	Reporting of the study	Dissemination of study findings^a^	Regulatory approval	Integration into guidelines	Adoption into practice	Evaluation of research
Funding of research	✓	✓	✓	✓					
Relationships with advocacy groups	✓				✓	✓	✓	✓	
Relationships with funding agencies	✓	✓							
Relationships with experts in the field		✓ Investigators	✓ Investigators	✓ Investigators, journal editors and peer reviewers	✓ Social media activists	✓ Systematic reviewers and panel members	✓ Systematic reviewers and panel members	✓	
Relationships with professional organisations	✓				✓		✓	✓	
Relationships with regulatory agencies and legislative bodies						✓			✓
Relationships with health practitioners	✓							✓	
Statements on research-related standards									✓

^a^Includes editorial, social media, press releases, media attention

### Funding of research

Fabbri et al. [14] proposed that industry sponsorship could bias the academic research agenda as a way to distract the public attention from the actual causes of health problems. They conducted a cross-sectional study on projects sponsored by the food industry and showed how research projects focused on a lack of physical activity (40%) compared to the consumption of highly processed foods (10%) as risk factors for health outcomes (e.g. obesity, diabetes) [14]. The study showed how corporations could bias the published evidence towards their interests and drive the research interests away from the most relevant public health problems to avoid blame for the public health problems [14, 15].

Furthermore, industry funding of trials could alter their design in favour of the sponsors [16]. A 2006 overview of clinical trials found that the majority of those funded by industry had, as a control arm, either placebo, a comparator drug belonging to the same company, or the same drug at a different dose [16]. Thus, the research agenda of each company is strongly focused on its own products and avoids testing them against existing drugs, which could be either equivalent or superior to their own.

There is also evidence of discrepancies between protocols and published reports of clinical trials sponsored by pharmaceutical companies, which compromises the credibility of their findings. Vedula et al. [17] found that internal company documents for gabapentin (e.g. statistical analysis plans, research reports) conflicted with the published research reports [17]. As an example, 3 out of 10 clinical trials had disagreement in the number of randomised patients between the internal reports and the publication (smaller number in the publications compared to the research reports) [17]. Those disagreements could undermine the transparency, accuracy and reliability of the published evidence.

Moreover, the funding of studies by corporations has been associated with findings favourable for that industry. A Cochrane systematic review found that studies sponsored by drug and devices companies are more likely than non-industry-sponsored studies to report favourable efficacy results (risk ratio (RR), 1.27; 95% CI 1.17–1.37; moderate certainty evidence) and to have favourable conclusions (RR, 1.34; 95% CI 1.19–1.51; low certainty evidence) [18]. The findings for harms were less conclusive (RR, 1.37; 95% CI 0.64–2.93).

Finally, industry involvement in research could affect the interpretation of results. Ebrahim et al. [19] found that authors of meta-analyses sponsored by the pharmaceutical industry are less likely to report negative statements regarding the assessed company’s drugs. A systematic review on nutrition studies found that studies sponsored by the food industry were more likely to have favourable conclusions compared to non-industry studies (RR, 1.31; 95% CI 0.99–1.72); however, the difference was not significant [20].

These examples show how research funding could affect critical steps of the research enterprise, including the choice of research topics, the design and conduct of the research, and the framing of its conclusions.

### Relationships with advocacy groups

It appears that the majority of advocacy groups accept substantive amounts of funding from industry [21]. Abola et al. [22] found that, out of 68 cancer patient advocacy organisations (PAOs)^1^, 75% disclosed a median of seven biopharmaceutical sponsors. Moreover, many of these PAOs promote drugs or procedures favouring their sponsors. The National Alliance of Mental Illness, for which the main funder is Pharma, pushed legalisation of certain drugs [23] and opposed the ‘black box’ warnings on antidepressants causing suicide and on attention deficit hyperactivity disorder drugs causing heart attack, stroke and sudden death [24]. Moreover, there is evidence that the majority of health advocacy organisations (HAOs)^2^ did not report on their source of funding [25]. For example, a study compared the Eli Lilly grant registry with the HAO websites and found that only a quarter of HAOs disclosed their funding by the company, and none disclosed the funding amount [25]. Advocacy organisations should be independent in their decisions and aware of their responsibility towards the people they represent; however, studies have shown that industry funding could affect their decision in favour of corporations’ interests [22, 24].

### Relationships with funding agencies

The industry could influence funders to such a degree that they affect the scientific strategy of regulatory organisations. Ong et al. [26] showed how the tobacco industry tried to redirect the funding of a second-hand smoke monograph by the International Agency for Research on Cancer through budgetary constraints of funders priorities. Nevertheless, the extent of such relationships is not clear as we have not identified other studies addressing this issue.

### Relationships with experts in the field

Industry–expert relationships might lead to bias in scientific research and evidence synthesis. A study examining systematic reviews on sugar-sweetened beverages found that authors with industry-related financial conflicts of interest (COIs) were five times more likely to report no positive association between the sugar-sweetened beverages and weight gain, compared to reviewers with no industry-related COIs [27]. Another review examined authors’ views in studies on rosiglitazone (antihyperglycemic drug) and the risk of myocardial infarction [28]. The review reported that authors with favourable recommendations for the drug were 4.3 times more likely to have financial COIs, particularly with the rosiglitazone manufacturer, compared to authors with unfavourable views [28].

The pharmaceutical industry–expert relationship might also influence the adoption of medications into practice, through medical education. On case study of a Canadian medical school found that lectures on pain pharmacotherapy were supported by the marketers of opioid analgesics and delivered by a member of the speakers’ bureaus of those companies [29].

### Relationships with professional organisations

The main role of professional organisations is to foster excellence and professionalism amongst its members. Industry relationships may undermine this role. There is evidence that the soda industry uses tactics to prevent any measures to discourage soda sales, including support of health professional organisations [30]. Sacks et al. [31] analysed email communications between the Coca-Cola executives and the leadership of the International Life Sciences Institute. They found that the industry affects the body of evidence through their influence on evidence generation and summation and over scientific bodies and medical associations, as well as through their relationships with policy-makers and opinion leaders [31]. A study of health organisations funded by Coca-Cola between 2010 and 2016 in Spain found that the majority of articles they published (70%) served the marketing strategy as they focus on physical inactivity as obesity risk factor instead of sugar consumption [32]. Similar concerns have been raised about how the pharmaceutical industry funds the activities of professional organisations, including research, annual meetings, clinical practice guidelines, training programmes and publications [33].

### Relationships with regulatory agencies

There is evidence of a growing influence of the pharmaceutical industry on the drug approval processes of regulatory agencies. ProPublica reported that the pharmaceutical industry funded 75% ($905 million) of the United States Food and Drug Agency’s (FDA) scientific review budgets for branded and generic drugs in 2017, compared to 27% in 1993 [34]. The key concern is that the regulatory agencies’ decision to approve certain medications might become biased in favour of the pharmaceutical industry.

Another industry strategy consists of after-the-fact compensation to those advising the United States FDA on drug approvals [35]. Out of 107 physicians advised by the FDA on drug approval, 66 (62%) received payments by drug companies (e.g. Brillinta), leading to what has been labelled ‘pay-later conflict’ [35]. A study found that 15 out of 55 of FDA’s haematology-oncology reviewers had later jobs or consultancies for the biopharmaceutical industry [36], creating bias among FDA employees through expectation of future employment [35]. In addition, former FDA employees recruited by pharma can exploit their relationships with former colleagues.

### Relationship with health practitioners

There is evidence that the relationships of physicians with the pharmaceutical industry affects their prescription behaviour. A review about the physician–pharmaceutical companies’ relationship in low- and middle-income countries found that the companies made visits to the majority of physicians (90%) and gave them gifts such drugs samples, simple gifts or sponsored items (e.g. travel) [37]. The review highlighted how these relationships could affect physician’s professional behaviour and prescribing habits [37]. Moreover, another study found that clinicians other than physicians (including nurses, nurse practitioners, physician assistants and pharmacists in the United States) had positive attitudes and favourable opinions towards industry interactions despite their knowledge about the competing interests [38]. Of note, these clinicians trusted the information from pharmaceutical representatives and enjoyed the “*easy access to information*” [39].

### Influencing research standards

The industry has made efforts to affect the standards of research transparency and integrity. One example relates to the Brussels Declaration, which attempts to provide guidelines “*for incorporating scientific progress into policy making*” [40]. McCambridge et al. [41] have demonstrated how the statement, under the influence of tobacco and alcohol industries, promotes industry involvement in incorporating science into policy-making without any explicit safeguards for the management of COIs. Interestingly, the sources of funding and support for the declaration itself were not reported [41]. Without clear rules organising industry involvement in knowledge and science generation, transparency and integrity would be compromised, not only in health research, but also in the standards regulating the research such as the Brussels Declaration.

Additionally, the pharmaceutical industry has had an impact on the regulatory standards by pushing for the elimination of “*unnecessary duplication of drug development and regulatory work*” [42], arguably compromising the drug-safety standards.

The industry could also influence the measuring tools used in research; for example, the tobacco industry made efforts to influence the global standards for measuring the tar content in cigarettes and which machines to use [43]. In 1998, Wigand argued that the methods used by the United States Federal Trade Commission underestimated the actual tar/nicotine deliveries from ventilated cigarettes by as much as 80%. He also reported that the Federal Trade Commission’s method was developed with significant input from industry. The above examples exemplify how the industry could affect the research standards and consequently affect the validity of findings and bias them in its favour.

Research standards are indirect elements of the research process; however, they affect the quality and integrity of the health research, such as entities’ COI disclosure policies and tar measurement devices, which may bias the body of evidence.

## Discussion

**Fig. 1:**
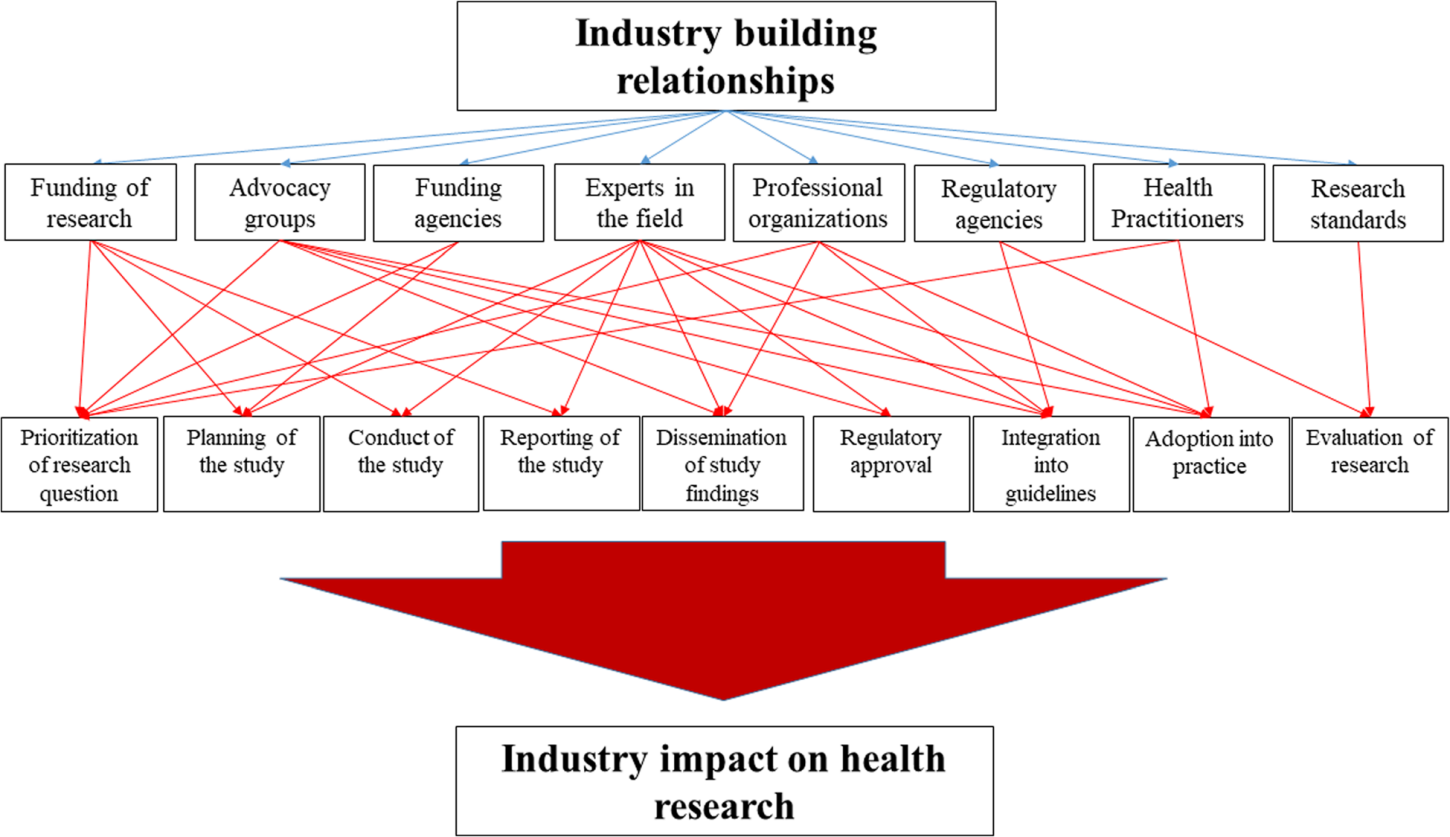
Figure illustrating how the industry impacts health research

The objective of this paper was to characterise the intersection between industry and the health research enterprise. We discussed eight types of relationships between the industry and the actors of health research, nine research steps of potential influence, and how these relationships allow the industry to exert influence. Figure 1 summarises how these concepts relate.

The industry has fostered relationships with almost every actor of the health research enterprise, ranging from advocacy groups to regulatory agencies. As detailed above, there is evidence that the industry has used these relationships to influence the different steps of health research, from prioritisation of the research question to the adoption of research findings into practice.

It is important to note that relationships with industry might have beneficial effects such as funding of educational programmes, rural development programmes and supporting research [10, 44]. Additionally, these relationships are not the only sources of bias in health research. For example, one has to consider financial COIs of experts that are not related to the industry (e.g. revenues related to patents, books, diet packages, innovative surgical techniques), as well as the non-financial COIs of those experts.

The degree of influence the industry is having on health research calls for more work on managing the relationships discussed here. Freudenberg and Galea [45] proposed a multifaceted response that included enhancing rights to information, restricting marketing, especially to children, constraining lobbying and sanctioning deliberate scientific distortions.

The right to information and transparency is an area that has gained significant momentum lately, with initiatives such as the Sunshine Act in the United States [46]. There have been calls for establishing a payment database for PAOs as an extension for the Sunshine Act law [47]. The North American Spine Society developed a disclosure policy for the organisation to regulate its relationships with industry funding and minimise industry influence and bias [48]. In addition, they expanded the disclosure policy to involve its members and published all members’ disclosures on its website [48]. Companies such as Coca-Cola have published their own databases and list of experts they worked with [49, 50]. However, investigators have shown that these databases are not comprehensive [51].

While disclosure of COIs has been the mainstay of managing some of the relationships (particularly with experts), Goldberg has argued that disclosure is at best ineffective, and likely harmful [52]; he called for a more conservative approach of sequestration, i.e. sequestering the relevant parties to the potential relationships.

Recently, Madureira Lima and Galea published a framework to “*systematically study corporations and other commercial interests as a distal, structural, societal factor that causes disease and injury*” [53]. Their framework aims to map corporate activities, and they briefly discuss “*control over the research process*” as one amongst many other factors.

It has become obvious that the industry has been very secretive and non-transparent in its strategies to increase its bottom line [54, 55]. Better characterising its intersections with the health research enterprise can help with better understanding its influence on that enterprise, and better structuring the debate around it. It can also help with organising the research in that field, both in terms of synthesising the existing literature and identifying the research gaps; in turn, this would guide the conduct of the primary research required for practice and policy in this area.

Further, there is a need for more research on how the industry’s influence on health research fits the wider context of the commercial determinants of health [9]. Indeed, the output of health research could be used at least in the first three of the four channels through which corporations exert their influence, according to Kickbusch [9], i.e. marketing, lobbying, corporate social responsibility strategies and extensive supply chains. Similarly, there is a need to better understand how the industry influences research in other fields, e.g. environment and development [56].

## Conclusion

It will be critical for the health research community to take on this agenda through strategic and well-organised efforts. One example is the Governance, Ethics and Conflicts of Interest in Public health (GECI-PH; twitter: @GeciPh) group, which is concerned with influence of industry funding on public health research, practice and policy outcomes. However, the success of such efforts will depend on the support of bodies invested in and entrusted with public health at the local and global levels.

Box 1 The types of relationships between the industry and the actors of health research 1. Funding of research

2. Relationships with advocacy groups

3. Relationships with funding agencies

4. Relationships with experts in the field

5. Relationships with professional organisations

6. Relationships with regulatory agencies

7. Relationships with health practitioners

8. Influencing research-related standards

Box 2 The relevant steps of health research 1. Prioritisation of research question

2. Planning of the study

3. Conduct of the study

4. Reporting of the study

5. Dissemination of study findings

6. Regulatory approval

7. Integration into guidelines

8. Adoption into practice

9. Evaluation of research

## Data Availability

Not applicable.

## Footnotes

1 Patient advocacy organisations: “*Patient advocacy organizations (PAOs) provide patient- and caregiver-oriented education, advocacy, and support services. PAOs are formally organized nonprofit groups that (a) concern themselves with medical conditions or potential medical conditions and (b) have a mission and take actions that seek to help people affected by those medical conditions or to help their families*” [57].

2 Health advocacy organisations: “*Health Advocacy Organizations (HAOs) are among the most influential and trusted stakeholders in US health policy, pursuing an agenda that includes expanding government support for medical research and the availability of health care services. In addition, HAOs advocate for members’ unrestricted access to all drugs, devices, and diagnostic tools relevant to their health conditions, almost always favoring branded drugs over generics, new screening technologies over older ones, and open formularies rather than closed ones*” [25].
